# Supplementary figures and images for: Burden of rare deleterious variants in WNT signaling genes among 511 myelomeningocele patients
Source: PLoS One. 2020 Sep 24;15(9):e0239083. doi: 10.1371/journal.pone.0239083 (PMC7514064; doi:10.1371/journal.pone.0239083)

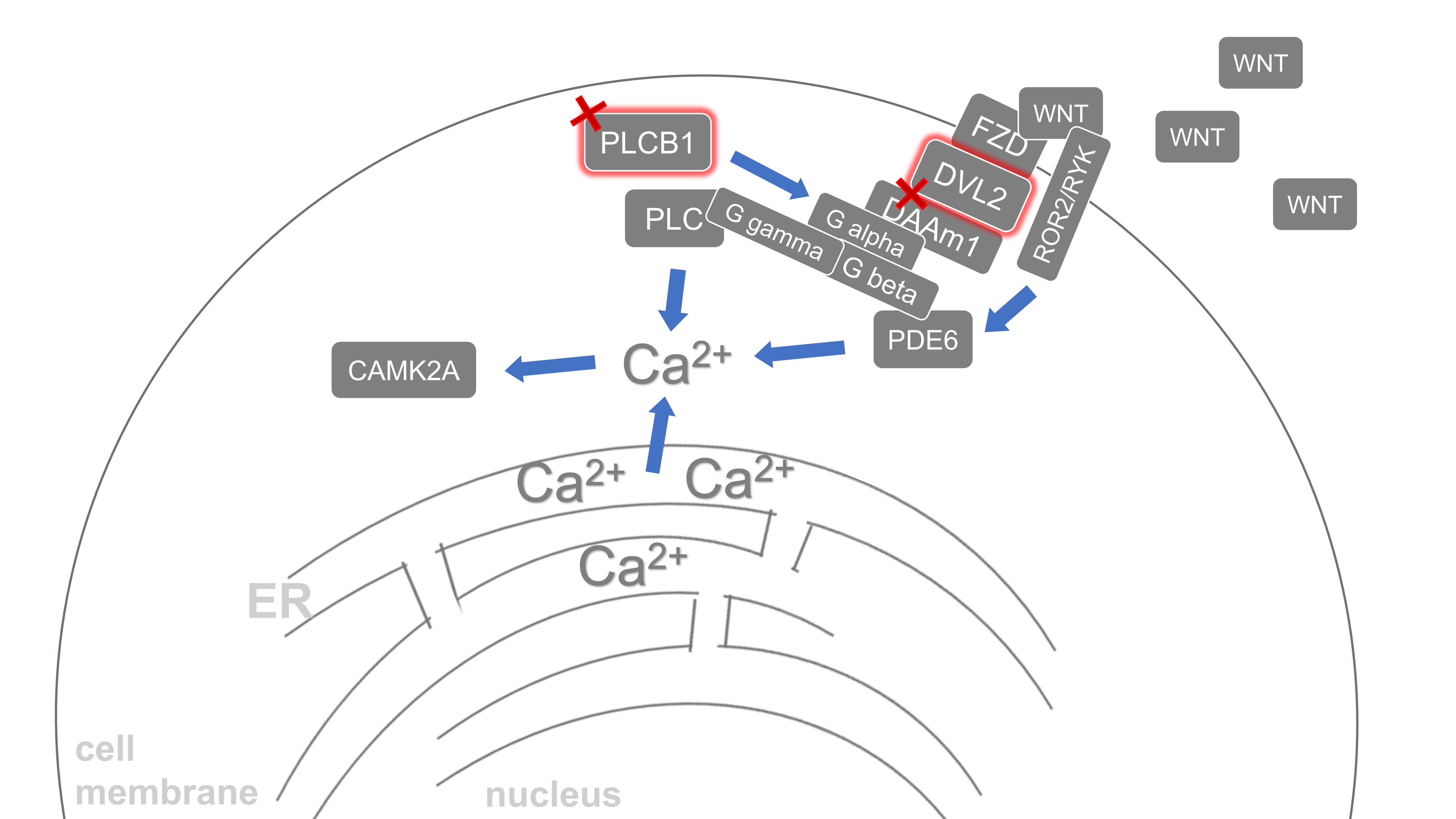

Supplement: S1 Fig — A depiction of PLCB1’s role in the noncanonical Ca2+ WNT signaling pathway. PLCB1 activates the G-protein coupled receptor’s alpha subunit, which is necessary for the downstream activation of CAMK2A. (TIF) [file pone.0239083.s001.tif]
