# Supplementary material for: Burden of rare deleterious variants in WNT signaling genes among 511 myelomeningocele patients
Source: PLoS One. 2020 Sep 24;15(9):e0239083. doi: 10.1371/journal.pone.0239083 (PMC7514064; doi:10.1371/journal.pone.0239083)
Supplement: Attachment — Submitted filename: Response to Reviewers_Hebert_wnt_text.docx [file pone.0239083.s008.docx]

Dear editor and reviewers,

We thank the reviewers for the comments and suggestions to improve the readability of our manuscript. We also thank the editor for the opportunity to submit a revised version of our manuscript for consideration to publish in PLOS ONE. Below are our responses to address the comments and concern from reviewers.

Best regards,

Kit Sing Au

**Responses to editor and reviewers’ commnets**

Journal Requirements:

When submitting your revision, we need you to address these additional requirements.

 1. Please ensure that your manuscript meets PLOS ONE's style requirements, including those for file naming. The PLOS ONE style templates can be found at

**Authors’ response: Thank you for the reminder. We have checked our manuscript to make sure it meets PLOS ONE’s style requirements.**

2. Please provide additional details regarding participant consent.

**Authors’ response: Lines 87 -92 in the “Changes” version, lines 71-76 of the “Manuscript” version, shows the ethics statements about written informed consent regarding participant of the study.**

Comments to the Author

1. Is the manuscript technically sound, and do the data support the conclusions? The manuscript must describe a technically sound piece of scientific research with data that supports the conclusions. Experiments must have been conducted rigorously, with appropriate controls, replication, and sample sizes. The conclusions must be drawn appropriately based on the data presented.
   a. Reviewer #1: Partly
   b. Reviewer #2: Yes
2. Has the statistical analysis been performed appropriately and rigorously?
   a. Reviewer #1: Yes
   b. Reviewer #2: I Don't Know
3. Have the authors made all data underlying the findings in their manuscript fully available? The PLOS Data policy requires authors to make all data underlying the findings described in their manuscript fully available without restriction, with rare exception (please refer to the Data Availability Statement in the manuscript PDF file). The data should be provided as part of the manuscript or its supporting information or deposited to a public repository. For example, in addition to summary statistics, the data points behind means, medians and variance measures should be available. If there are restrictions on publicly sharing data—e.g. participant privacy or use of data from a third party—those must be specified.
   a. Reviewer #1: Yes
   b. Reviewer #2: Yes
4. Is the manuscript presented in an intelligible fashion and written in standard English? PLOS ONE does not copyedit accepted manuscripts, so the language in submitted articles must be clear, correct, and unambiguous. Any typographical or grammatical errors should be corrected at revision, so please note any specific errors here.
   a. Reviewer #1: Yes
   b. Reviewer #2: Yes
5. Review Comments to the Author. Please use the space provided to explain your answers to the questions above. You may also include additional comments for the author, including concerns about dual publication, research ethics, or publication ethics. (Please upload your review as an attachment if it exceeds 20,000 characters)
   a. Reviewer #1:
      i. Summary: The authors of this manuscript have identified that specific genes in the WNT signaling pathway have a higher mutational burden in terms of the pathogenesis of myelomeningocele, a neural tube defect in humans. The authors developed an extensive analysis pipeline to uncover candidate genes. Ten genes were discovered that were primarily important for planar cell polarity with higher mutational burden in the population of Mexican Americans, and seven genes with higher mutational burden in the population of European ancestry. Then the authors performed GO analysis and established the relationship between the rare deleterious variants (RDVs) in genes within different aspects of the Wnt signaling pathway. This study may be helpful to understand the genetic pathogenesis of human neural tube defects.
      ii. There are two major caveats: one, the same rare mutations are also found in control populations and two, the absence of experimental evidence that these mutations damage biological functions. This weakens the conclusions for pathogenesis of these variations.

**Authors’ response: We thank the Reviewer for the comment and we also thank the editor for clarifying that we do not need to address these two concerns.**

iii. Major concerns:
     1. In the introduction, the authors could better outline their thought process of how they went from stating that there are 372 disrupted genes in mouse models of NTDs, to saying they suspect these deleterious mutations for human NTDs could be in the WNT signaling pathway above any other cell signaling pathway important for development.

**Authors’ response: The beginning of the Introduction’s third paragraph (lines 63 to 70) has been modified to help bridge this gap.**

2. In the current cases, are there clinical phenotypes other than myelomeningocele?

**Authors’ response: Only isolated myelomeningocele diagnoses were included in the study and the first paragraph in the Methods section has now been amended to clarify that information. More details about patient recruitment can be found in the Au *et al*. 2008 study cited both in the manuscript and below.**

a. **Kit SA, Tran PX, Tsai CC, O’Byrne MR, Lin JI, Morrison AC, et al. Characteristics of a spina bifida population including North American Caucasian and Hispanic individuals. Birth Defects Res Part A - Clin Mol Teratol. 2008;82(10):692–700.**

3. If possible, please describe the biological context of the gnomAD controls. Were they overall “healthy” with no other health conditions?

**Authors’ response: gnomAD’s “controls” populations only include individuals recruited to studies as controls. In other words, these individuals did not have whatever disease of interest the original studies investigated. Also, the gnomAD data has been vetted not to include severe pediatric disease or relatives of anyone with a severe pediatric disease. gnomAD’s website reads “We have removed individuals known to be affected by severe pediatric disease, as well as their first-degree relatives, so these data sets should serve as useful reference sets of allele frequencies for severe pediatric disease studies - however, note that some individuals with severe disease may still be included in the data sets, albeit likely at a frequency equivalent to or lower than that seen in the general population.” (**<https://gnomad.broadinstitute.org/about>**)**

a. **To minimize any reader confusion, we have replaced the phrase “gnomAD controls,” for “gnomAD references” in the manuscript.**
4. Page 10 line 211, what do the authors mean by two European populations? Please describe the two populations clearly.

**Authors’ response: This sentence was meant to read “populations of European ancestry.” The sentence has been corrected.**

5. How do the authors explain that nominally significant genes associated with myelomeningocele do not overlap in the EA and MA populations?

**Authors’ response: The 2019 gnomAD article establishes that the EA and MA populations are largely nonoverlapping in a 10-part principal component analysis (see their figure 1A). Knowing that these populations have unique patterns of variation, at least two explanations are possible. First, biological processes such as WNT signaling often require the function of many gene products. Losing function of any key players may lead to the same result of disrupting normal WNT signaling, even if different key gene players are lost in each population. This possibility is now included in the second paragraph of the Discussion’s Gene Ontology Enrichment Analysis section (lines 444-446). Alternatively, different biological mechanisms could explain the non-overlapping genes. This possibility has been emphasized in paragraph 3 of the Discussion’s Gene Ontology Enrichment Analysis section (lines 449-450).**

**a. Karczewski KJ, Francioli LC, Tiao G, Cummings BB, Alföldi J, Wang Q, et al. Variation across 141,456 human exomes and genomes reveals the spectrum of loss-of-function intolerance across human protein-coding genes. bioRxiv [Internet]. 2019; Available from:** [**https://www.biorxiv.org/content/10.1101/531210v2**](https://www.biorxiv.org/content/10.1101/531210v2)

6. Provide a table of the 17 genes indicating which ones are heterozygous and which ones are homozygous in the patients. How many frameshift mutations were found in the present cohort? Were they included in the statistical analysis?

**Authors’ response:**

a. **Because all but one of the 17 genes contained only heterozygous variants, we thought a written summary would communicate this information well. The third paragraph of the Results section (lines 218-219) now starts with a sentence summarizing heterozygous versus homozygous information of the nominally significant genes.**
b. **As described in the Materials and Methods section, we only evaluated single nucleotide variants that caused non-synonymous substitutions, stop-gained, stop-lost or splice sites changes in this analysis. The database for non-synonymous functional predictions (dbNSFP) which we used to annotate our variants with CADD phred scores and gnomAD control data, only provides information for non-synonymous substitutions. In addition, quality control filtering proved to be untenable for indels after a test using sanger sequencing. Therefore, frameshift variants from indels were excluded from the statistical analyses of this study.**
7. Fig. 2 indicates there may be more than one Wnt pathway gene that contains an RDV in an individual case. Please include a table that shows which genes (2 or more) that contain RDVs in an individual. Also include whether the NTD is more severe or associated with other phenotypes in these cases with multiple Wnt RDVs. Are multiple Wnt RDVs observed in controls? Do the multiple RDV mutations affect proteins within a complex or do they hit different aspects of the Wnt pathway?

**Authors’ response:**

a. **In the third paragraph of the results section (lines 224-227), we have now listed all combinations of genes where two or more nominally significant genes contained a rare deleterious variant in a single patient sample. Also, a supplemental file has been added that includes these combinations (Table S7). Figure 2 does indicate there are individual cases carrying more than one gene containing an RDV that qualified for mutation burden analysis. However, since this study focuses on whether there were some genes with higher mutation burden in the case cohorts, the significance of the occurrence of multiple RDV-containing genes per individual case is out of the scope of the current analysis. In addition, as explained below in (c.), we cannot perform a statistical analysis to compare individual cases with individual gnomAD references. Therefore, it is prudent for this study to avoid presenting cases with RDVs and RDVs-containing genes that did not pass nominally significance on mutational burden test.**
b. **All our cases are affected with myelomeningocele with lesions spanning from the lower T vertebrae (T11, T12) to the sacral vertebra. Other phenotypes, such as mobility, were not used because the outcome can be affected by the degrees of available rehab or medical interventions. Attempts to correlated genotypes and lesion levels by counting the vertebra affected were not fruitful.**
c. **gnomAD does not make individual-level genotype data available, only populational data. Although individual genotypes of gnomAD data may be retrievable through dbGaP, this would require approvals of data usage from individual projects contributing to the gnomAD_controls datasets with different data usage restrictions and is not guaranteed from all control data analyzed.**
d. **The multi-gene hits only occur in genes whose protein products do not directly complex with one another. String-db.org helped confirm this. However, some proteins are involved in the same Wnt signaling sub-pathways, such as CPE & PORCN, PTPRU & PSMD3, PTPRU & FERMT2, and FERMT2 & PSMD3 (see figures 3 & 5). As shown in S7_Table, the myelomeningocele phenotypes for the five MA subjects having two nominally significant RDV-containing genes have no remarkable differences compare to other subjects in the study.**
8. It looks like all RDVs reported in the present cohorts could be found in control population, and there are no functional experiments to demonstrate the RDVs can disrupt the biological functions, so it is problematic to evaluate the contribution of current RDVs to the pathogenicity of myelomeningocele.

**Authors’ response:**

a. **We thank the Reviewer for the comment and we also thank the editor for clarifying that we do not need to address the concerns about RDVs present in control (reference) population and no functional experiments were presented for these variants. There are RDVs that are not present in the reference populations as shown in S5 and S6 Tables. In addition, the alternate allele frequencies observed in cases were generally one log higher than those found with the reference populations (median of 10^-4^ for MA and 10^-5^ in EA). The burden analyses approach was used in this manuscript to specifically differentiate whether cases had higher burden of RDVs in some of their WNT signaling genes contributing to increase risk of developing myelomeningocele.**
9. Regarding the GO analysis, since the basis of the study is to examine genes functioning in the WNT signaling pathway, including core genes and associated genes, it is unreasonable to do GO analysis and draw conclusion on the WNT signaling pathway, although the deeper analysis within the pathway relative to population RDV is OK. However, some genes like DVL2, which is a hub of Wnt signaling and is involved in both canonical and non-canonical Wnt signaling, are categorized in Table 2 as only within the canonical signaling pathway.

**Authors’ response: We thanks the reviewer for the comments.**

a. **We try to explain in the first paragraph of the Discussion’s Gene Ontology Enrichment analysis section that all terms synonymous to “Wnt signaling” are due to our limited initial Wnt signaling 523-gene set. However, we look past these umbrella terms for any categories that define a more specific arm within the pathway. The more specific terms help guide our understanding of which disrupted parts of overarching Wnt signaling might be to blame for myelomeningocele.**
b. ***DVL2* is included under the Gene Ontology term “non-canonical Wnt signaling pathway” (**[**http://amigo.geneontology.org/amigo/term/GO:0035567**](http://amigo.geneontology.org/amigo/term/GO:0035567)**). However, the non-canonical Wnt signaling term did not come out when conducting an enrichment analysis because the list of genes queried did not reach the statistical threshold to demonstrate an enrichment for non-canonical Wnt signaling. Table 2 only shows *DVL2* under its canonical Wnt signaling label because the disrupted gene set was enriched for genes involved in canonical Wnt signaling but not enriched for genes involved in non-canonical WNT signaling.**
10. How were miRNA binding sites in the genome defined?

**Authors’ response:**

**The miRNA binding sites were predetermined by the proprietary exome capture library kit manufactured by LifeTechnologies base on published information and data from miRNA target databases (e.g. miRbase and miRBD). Our study focuses on the exons of protein coding genes and miRNA binding sites are typically found in 3’ UTRs, so variants involving miRNAs were excluded from our analysis. However, an analysis including miRNA variants is a relevant and interesting future direction.**

11. Line 240, please explain SCF-KIT signaling.

**Authors’ response:**

**We agree that the mention of SCF-KIT as well as the terms dealing with myoblasts did not add substance to the results or discussion. Mention of these terms was removed from the Discussion’s Gene Ontology Enrichment Analysis section, 2^nd^ paragraph (line 436) and the last paragraph of the Results (line 239) of the original manuscript file.**

12. How do the authors consider the presence of RDV mutations in genes that are not detected in the neural tissue of humans (Carnegie stage 12 and stage 13, as cited)?

**Authors’ response:**

**The SAGE data is specific but it has a detection limit like all other experimental tools. More sensitive tools such as microarray or RNAseq methods may better define the expression profiles. So, the absence of a gene’s detection in that dataset is not strong evidence that the gene is not expressed. However, detection of a gene is suggestive that the gene is expressed in human neural tubes during the period of neural closing and the result was observed from multiple samples.**

13. The OR value may not be appropriate to evaluate risk of disease, as the number of control and case are relatively mismatched.

**Authors’ response:**

**We appreciate the comment. As presented in the manuscript Materials and Methods, the Fisher test *P* values were used as primary tool to guide our observation and discussion. Presenting the OR values is a supplement to help reader further understand the confidence interval ranges for these analyses.**

14. Some papers have already reported the genetic contributions of genes functioning in Wnt signaling pathways to neural tube defects, please summarize them in the discussion and clarify the ethnicity feature of genetic mutations of Wnt signaling pathway in human neural tube defects.

**Authors’ response:**

**We have added subheading “Perspective” and a sentence in the Discussion section (lines 459-line 471 of the “Manuscript”; lines 499-503 of the “Changes” version) to summarize a recent review that evaluates rare deleterious variants associated with NTDs in humans. While explaining the difference others’ findings and our own, we highlight the likely important factor of ethnicity.**

iv. Minor concerns:
    1. Lines 240-243 please expand on these signaling pathways mentioned and how they are related to the WNT signaling pathway genes being affected. If they aren’t related, state that or take it out of this section and put it in the discussion where you can discuss their possible importance.

**Authors’ response: Please see our response to comment #10.**

2. is there a DDB1 mouse mutant? What is its phenotype?

**Authors’ response:**

**Yes, mice completely null for *Ddb1* are embryonic lethal and those with conditional inactivation of *Ddb1* have CNS issues. No information was presented on neural tube morphology except reporting extensively degenerated embryos at E12.5. We added this information in another sentence in the third paragraph of the Discussion’s section on “Myelomeningocele exomes with canonical WNT/β-catenin pathway genes disrupted” (lines 397-400).**

3. Page 5 line103 and 107, “S1” should be corrected to Table S1?

**Authors’ response: All supplemental files previously labeled “Text” have now been changed to “Table.”**

4. Page 7 line 150, a CADD paper should be cited here.

**Authors’ response: Yes, we have now included this citation.**

5. In Table 1 “CI” means 95% CI or something else?

**Authors’ response: Yes, “CI” = confidence interval (see the Table 1 figure description, lines 208-209)**

b. Reviewer #2:
   i. The authors hypothesized that some genes in the WNT signaling network have a higher mutational burden in myelomeningocele (MMC) subjects than in controls. Exome sequencing was performed from 511 MMC was performed and data from 29,940 ethnically matched subjects was provided by version 2 of the publicly available Genome Aggregation Database. To compare mutational burden, the authors searched rare deleterious variants across each of 523 human WNT signaling genes in case and control populations. 10 WNT signaling genes were disrupted with a higher mutational burden among Mexican American MMC subjects compared to controls and 7 genes were disrupted among individuals of European ancestry compared to controls. Gene ontology enrichment analyses indicate that genes disrupted only in the Mexican American population play a role in PCP pathway whereas genes identified in both populations are important for the regulation of canonical WNT signaling. The authors presented an impressive amount of data on a very large group of patients and mutational burden analysis provides more power than single-variant approach.

**Authors thank reviewer for comments.**

6. PLOS authors have the option to publish the peer review history of their article (what does this mean?). If published, this will include your full peer review and any attached files. If you choose “no”, your identity will remain anonymous but your review may still be made public. Do you want your identity to be public for this peer review? For information about this choice, including consent withdrawal, please see our Privacy Policy.
   a. Reviewer #1: No
   b. Reviewer #2: No
